# Outcomes and Utilization of a Low Intensity Workplace Weight Loss Program

**DOI:** 10.1155/2014/414987

**Published:** 2014-01-02

**Authors:** Kelly M. Carpenter, Jennifer C. Lovejoy, Jane M. Lange, Jenny E. Hapgood, Susan M. Zbikowski

**Affiliations:** ^1^Alere Wellbeing, 999 Third Avenue, Suite 2100, Seattle, WA 98104, USA; ^2^University of Washington, Seattle, WA 98195, USA

## Abstract

Obesity is related to high health care costs and lost productivity in the workplace. Employers are increasingly sponsoring weight loss and wellness programs to ameliorate these costs. We evaluated weight loss outcomes, treatment utilization, and health behavior change in a low intensity phone- and web-based, employer-sponsored weight loss program. The intervention included three proactive counseling phone calls with a registered dietician and a behavioral health coach as well as a comprehensive website. At six months, one third of those who responded to the follow-up survey had lost a clinically significant amount of weight (≥5% of body weight). Clinically significant weight loss was predicted by the use of both the counseling calls and the website. When examining specific features of the web site, the weight tracking tool was the most predictive of weight loss. Health behavior changes such as eating more fruits and vegetables, increasing physical activity, and reducing stress were all predictive of clinically significant weight loss. Although limited by the low follow-up rate, this evaluation suggests that even low intensity weight loss programs can lead to clinical weight loss for a significant number of participants.

## 1. Introduction

Obesity affects over one third of the US population [1], and obesity-associated health conditions result in excess economic costs of $147 billion per year in the United States [2]. In addition to direct healthcare costs from obesity, current estimates of financial losses due to obesity-related absenteeism and presenteeism (low productivity while on the job) are currently in the range of $73 billion annually [3]. Projections from a recent report [4] indicate that by 2030 medical costs associated with treating preventable obesity-related diseases could increase by $48–$66 billion per year, and the loss in economic productivity could be between $390 and $580 billion annually.

Health problems related to obesity include cardiovascular disease, diabetes, hypertension, cancer, kidney disease, strokes, osteoarthritis, and sleep apnea. Furthermore, obese individuals suffer from stigma, which leads to decreased quality of life [5] and healthcare disparities, resulting in, for example, fewer cancer screenings [6]. With even a modest weight loss of 5% of body weight, quality of life can improve, many health conditions can be prevented or ameliorated, and healthcare and productivity costs can be contained [4, 7, 8].

Behavioral treatment for obesity, including monitoring weight and food, caloric restriction, and increased physical activity, has been shown to be effective for long-term weight management and prevention of chronic disease. For example, the Diabetes Prevention Program (DPP) evaluated intensive lifestyle counseling compared with medication and usual care. The DPP intensive lifestyle counseling arm included 16 one-on-one in person coaching sessions and additional group meetings and resulted in a 4-year sustained weight loss and a 58% reduction in diabetes incidence, outperforming both the medication condition and the control condition [9]. Similar long-term weight loss and disease prevention benefits from intensive lifestyle treatment have been shown in the Look AHEAD trial, the Finnish Diabetes Prevention Study, and the Chinese Da Qing diabetes prevention study [10–12]. Thorpe and Yang [13] advocate for the dissemination of weight loss programs based on the lifestyle intervention developed for DPP and project billions of dollars saved if such programs were to be implemented at a national level.

Unfortunately, a high intensity, face-to-face intervention such as the DPP is difficult and expensive to disseminate. Thus, a variety of modestly successful attempts have been made to preserve outcomes, while decreasing participant burden and program costs [14]. One method of controlling costs and decreasing participant burden is the use of the telephone counseling. Telephone-based weight loss counseling has been shown to be as effective as face-to-face interventions [15]. Furthermore, phone-based counseling is highly cost effective [16] and reaches more underserved populations than traditional clinic-based methods [17, 18]. The Internet also provides an alternative method of low intensity weight loss treatment dissemination [19]; however, internet-only weight loss programs are generally less effective than phone or in-person programs [20, 21].

Workplace-based or employer-sponsored weight loss programs, often provided as a benefit to employees by employers who seek to increase the health and productivity of their workforce and decrease their health care costs, have been found to be acceptable by workers and modestly effective for reducing body weight [22, 23]. Workplace weight loss interventions vary widely in their offerings and may include on-site physical activity options, monetary incentives, nutrition information, social support, and/or behavioral counseling [22].

While there have been a number of recent peer-reviewed publications showing the outcomes of workplace weight loss programs, few have examined the effect of employer-sponsored integrated phone- and internet-based weight management coaching and none to our knowledge have examined the impact of treatment utilization on health behavior changes and outcomes. The present paper describes the weight loss outcomes, health behavior changes, and program utilization of an employer-sponsored low intensity phone- and web-based weight loss program and examines independent predictors of weight loss.

## 2. Method

### 2.1. Recruitment and Enrollment

Employees from fifteen employers were offered access to an integrated phone- and web-based weight management program as an employee benefit. These employers included a range of occupations, both blue collar and white collar. All employees were informed about the program in their worksite and invited to enroll via a website. Worksites promoted the program with emails, posters, and fliers. The enrollment process consisted of web-based collection of demographic and baseline information, determination of eligibility, and receipt of a Health Insurance Portability and Accountability Act (HIPAA) and privacy notice form.

To be eligible for the weight loss program, employees had to be over the age of 18 and fluent in spoken and written English. Current pregnancy, bariatric surgery (past or planned in next six months), and eating disorder history (anorexia and bulimia) were exclusions. Body Mass Index (BMI) of 25 or higher (e.g., BMI > 25 kg/m^2^) was required for inclusion in this dataset as a BMI over 25 has been associated with a variety of negative health consequences. The present study received a waiver of consent from the Institutional Review Board of record.

## 3. Intervention

The workplace weight loss and wellness program was based on the National Institute of Health (NIH) Clinical Guidelines on Identification, Evaluation, and Treatment of Overweight and Obesity in Adults [24] and included nutrition recommendations based on the NIH-developed “Dietary Approaches to Stop Hypertension” eating plan [25] and physical activity recommendations from the American College of Sports Medicine and the Physical Activity Guidelines for Americans developed by the US Department of Health and Human Service [26]. Because it has been well demonstrated that maintaining weight loss, rather than initially losing the weight, is the most difficult part of weight management, the weight loss program made use of the substantial evidence-base of studies on what behaviors (high levels of physical activity, stress management, and lifelong healthy eating patterns) are necessary to prevent relapse and weight regain [27–30].

Participants in the weight loss program were offered three proactive phone-based counseling sessions (initiated by the program staff), two with a weight loss health coach, and one focused on nutrition with a registered dietician (RD). Calls were made during predetermined times at the participant's convenience. If the coach could not reach the participant, five additional attempts were made to reach the participant by telephone and emails were sent to the participant to encourage reengagement in the program. In addition to the scheduled outbound calls from the coaches and RDs, participants could call into the program to talk to a coach or RD at any time during program participation. The telephonic coaching sessions involved a health assessment, tailoring dietary and physical activity recommendations, and motivational and cognitive behavioral interventions such as increasing self-efficacy, problem solving, and goal setting. Eating more servings of fruits and vegetables, eating breakfast every day, increasing physical activity up to 180 minutes per week, and reducing stress were all emphasized as healthy behaviors. Program participants were also offered four survey calls. The program also included an integrated website, a comprehensive resource offering eLearning modules for core skills and health behaviors such as stress reduction, time management, improving body image, and coping with difficult eating situations. Other features included a moderated online support community, ability to email or chat with a coach, and tracking tools. Participants had lifetime access to the website as long as they remained employees of one of the eligible companies. Participants also received a welcome kit which included a program guidebook, a pedometer, a tape measure for assessing waist circumference, and a food journal.

## 4. Measures

Baseline data were collected via the web at program registration and by phone during the first coaching call. Six and twelve-month follow-up data were collected via survey calls conducted by trained evaluation specialists. In addition, login and weight and activity tracker use data from the website were collected. Outcome measures included self-reported body weight and height from which BMI (kg/m^2^) was calculated. A subset of 167 participants provided self-reported blood pressure. Participants also reported frequency of key health behaviors that were emphasized in the program: breakfast consumption (days/week), fruit and vegetable consumption (servings/day), physical activity (days/week), and stress (using a four-point scale ranging from 1 = no stress to 4 = extreme stress). Our primary outcome was clinically significant weight loss at six months, defined as a loss of 5% or more of initial body weight [8].

Program utilization was measured by total number of coaching telephone calls and website logins. Website tool utilization was measured by number of uses of the step counter, activity tracker, and weight tracker tools. Use of the online support community and email/chat with a coach was very limited and, thus, use of the these features was not included in the analysis. Sociodemographic and baseline health variables included age, gender, and chronic disease history (i.e., hypertension, diabetes, high cholesterol, and mental health condition).

## 5. Statistical Analysis

Descriptive statistics were used to characterize baseline demographics, weight loss outcomes, treatment engagement, and behavioral changes for six-month and twelve-month responders.

We examined three multivariate logistic regression models to determine predictors of clinically significant six-month weight loss in terms of program utilization and behavioral changes. The first model looked at program utilization (number of calls and number of web logins) as independent predictors of clinically significant weight loss. The second looked at utilization of specific web features by replacing web logins with use of three tracking tools. The third model looked at changes in recommended health behaviors as predictors of weight loss. All models controlled for baseline demographic and health variables, including age, gender, baseline BMI, baseline stress, and history of chronic disease (*yes* or *no* to any). The alpha level for significance was set at *P* < 0.05. Due to the exploratory nature of study, we did not include corrections for multiple comparisons. Interpretation of all odds ratios in these models assumes all other variables in the model are held constant.

## 6. Results

### 6.1. Participants

Between 2009 and 2011, 2,917 individuals enrolled in the program with the stated reason of losing weight and who had a BMI of 25 or higher. Of these, 1,070 (37%) completed the follow-up survey at 6 months, and 687 (24%) completed the survey at 12 months. A total of 473 had both 6- and 12-month data. In our sample, male participants and those with a history of chronic disease had higher rates of completing the follow-up surveys.

### 6.2. Baseline Characteristics

Overall, 40% of those who responded to the 6-month survey had a starting BMI of 25–30 kg/m^2^ (overweight) and 60% had a BMI greater than 30 (obese). Average BMI at program entry was 32 kg/m^2^ (SD 10.8). Women comprised 54% of this sample. The mean age was 43 years (SD 5.1). Fifty-six percent had at least one chronic health condition, the most common conditions being high blood pressure (22%), high cholesterol (29.7%), and mental health conditions (21.1%).

### 6.3. Weight Loss and Blood Pressure Outcomes

Among those who responded to the 6-month survey, 34% (*n* = 363) lost at least 5% of their body weight from baseline, and 11% (*n* = 113) had lost at least 10%. On average, participants lost 7.8 pounds (SD 11.4) and 4.2% of their original body mass. Of the 687 participants reporting at twelve months, 39% (*n* = 269) had lost at least 5% of their body weight, and 16% (*n* = 112) had lost at least 10%. Mean 12-month weight loss was 9.2 lbs (SD 14.3).

Because weight loss is known to be associated with reductions in health risk factors, we analyzed changes in blood pressure for those participants who self-reported this measure. Participants with blood pressure (BP) measures at baseline and 6 months (*n* = 167) had average reductions in systolic BP of 2.6 mmHG (SD 11.6) and reductions in diastolic BP of 1.4 mmHG (SD 8.4).

### 6.4. Program Utilization

On average, 6-month responders engaged in 3.1 total coaching calls (SD 1.2), including an average of 2.5 (SD 0.7) of the 3 scheduled outbound calls and 0.6 (SD 0.6) support calls. Total number of calls ranged from 1 to 5. Summary statistics regarding the use of web tools are summarized in Table 1 with medians and interquartile ranges (IQR). The distributions of usage counts for each of these tools were positively skewed: most participants used the tools relatively few times, but some used them hundreds of times.

### 6.5. Health Behavior Change

At baseline 19% of the 6-month responders were eating at least 4 servings of fruits and vegetables per day, 51% were eating breakfast 6 to 7 days per week, and 35% were physically active at least 4 days per week. Thirty-nine percent of the sample reported “quite a lot” or “extreme” stress at baseline. At 6-month followup, 60% of respondents reported eating at least 4 servings of fruits and vegetables per day, 65% were eating breakfast 6 to 7 days per week, and 62% reported physical activity at least 4 days per week. Overall, 61% of 6-month responders reported increasing fruit and vegetable consumption, 35% reported increasing breakfast consumption, and 49% increased their physical activity. Twenty-eight percent reported decreasing their stress level.

### 6.6. Predictors of Clinically Significant 6-Month Weight Loss

#### 6.6.1. Program Utilization: Coaching Calls and Website Logins

The first multivariate logistic regression model considers the contributions of the two main clinical aspects of the program: coaching calls and website use. Adjusting for baseline covariates, both the number of coaching calls and the number website logins were independently predictive of ≥5% weight loss at six months (Table 2). Adjusting for other factors, each additional coaching call was associated with an odds ratio (OR) of 1.16 (95% CI (1.03, 1.30), *P* = 0.016), indicating a 16% increase in odds of ≥5% weight loss for each additional call. Each additional website login was associated with an OR of 1.009 (95% CI (1.004, 1.01), *P* < 0.001).

### 6.7. Specific Web Tools

The next model considers the contribution of specific web tools to ≥5% weight loss. Controlling for baseline variables and coaching calls, each additional use of the weight tracking tool was associated with an OR of 1.04 (95% CI (1.02, 1.07), *P* = 0.002; Table 3). In this model use of neither the activity tracker nor step tracker was independently predictive of weight loss; nor were they jointly predictive (likelihood ratio *P* = 0.59). The estimated OR for coaching calls was attenuated compared to the first model (OR = 1.11, 95% CI (0.98, 1.26), *P* = 0.084).

### 6.8. Health Behavior Changes as Predictors of ≥5% Weight Loss

The next model considers how improvements in health behavior predicted ≥5% weight loss at six months, controlling for baseline factors but not program usage. Adjusting for other factors, improvements in fruit and vegetable consumption were associated with clinically significant weight loss (OR = 1.45, 95% CI (1.08, 1.94), *P* = 0.013), as was an increase of days of physical activity (OR = 1.38, CI (1.05, 1.80), *P* = 0.022), and an increase in the number of days per week of eating breakfast (OR = 1.35, 95% CI (1, 1.81), *P* = 0.047; Table 4). A decrease in stress levels from baseline to 6-month followup was also associated with weight loss (OR =1.53, 95% CI, (1.07, 2.19), *P* = 0.021).

## 7. Discussion

The current study indicates that a low intensity weight loss program that offers a relatively low number of behavioral counseling calls combined with web resources can lead to clinically significant weight loss for about one third of participants who responded to the follow-up survey at 6 and 12 months. Participation in the program, both number of counseling calls and website logins, independently predicted clinically significant weight loss. Positive health behavior changes were evident even in those who did not lose a clinically significant amount of weight, with nearly two thirds of participants increasing fruit and vegetable consumption and nearly half increasing physical activity by 6 months. About 30% of participants also reduced their stress levels from baseline to six months. Although the majority of participants saw improvements in health behavior, improvements were greater among those with clinically significant weight loss. Overall, at 6 months, a majority of participants were eating at least four servings of fruits and vegetables each day, eating breakfast most days, and reporting at least four days per week of physical activity.

Of the web tools, use of the weight tracker was the most significant predictor of weight loss and appeared to be associated with weight loss independent of possible effects on specific health behaviors. Other studies examining use of weight loss websites have also found that use of a self-monitoring tool for weight is related to weight loss, while other features seem less predictive [31]. This is consistent with a number of previous studies showing that regular self-monitoring of weight is a critical factor in weight loss and maintenance [32]. We did not find that activity or step tracking tools predicted weight loss after adjusting for other factors, but actual use of these tools was quite limited.

Not surprisingly, the nutrition-related health behavior changes (increasing consumption of fruits and vegetables and daily breakfast) also predicted weight loss. What was somewhat unexpected is that increase in physical activity also predicted loss, although use of activity tracking tools was not predictive. It is well known that physical activity alone has little impact on weight and little added benefit to dietary restriction in behavioral weight loss programs [33]. Physical activity, however, remains an important part of weight loss programs as it is critical for weight loss maintenance, improves overall health and wellbeing, and plays a role in preserving lean body mass during weight loss [34].

One of the most interesting findings was that those participants who were able to reduce their stress during program participation were more likely to lose at least 5% of their body weight. Due to the nature of the study, it is not known how participants decreased their stress, whether it was purposeful, nor can we determine whether lowering stress had a causal relation to weight loss. However, high levels of psychological stress have been implicated in weight gain, failure to adhere to weight loss programs, and failure to maintain weight loss [35–37]. Acute stress can trigger increased appetite and overeating [38–41] and may lead to higher likelihood of storage of fat in the abdomen which has negative health consequences [39]. Furthermore, foods consumed during stress often contain high levels of sugar and fat, increasing the likelihood of weight gain [42]. Although stress management is often included as a minor element of weight loss programs, there has been a recent call for more emphasis to be placed on this construct [43].

Although limited to a small subset, the reported decrease in blood pressure is clearly important in terms of reducing healthcare costs and chronic disease burden. A drop in average blood pressure of 5 mm Hg is associated with a 34% decrease in risk for stroke and a 21% decrease in ischemic heart disease [44]. While weight loss is clearly an important factor in reducing blood pressure, the dietary component of the program utilized the DASH eating plan, which has also been shown to reduce blood pressure independent of changes in weight [25].

This research does have limitations. First, this was not a randomized study. The group was self-selected and encouraged by their employers which may limit the generalizability of results on program effectiveness and weight loss. Another significant limitation of this study is that only 37% of original participants responded to the six-month follow-up survey. It is not clear how many of those who did not respond to the survey had dropped out of the intervention as opposed to being involved but not responding to survey calls. If survey respondents differed systematically from the nonrespondents with regard to weight loss, our findings may not be broadly generalizable. High drop-out rates are common in commercial weight loss programs [45]; however, the higher rates of followup in research studies may be an artifact of the high bars set for participation and compensation for assessment. Another limitation is the lack of verification of body weight through biometrics: all data collected were self-reported.

We were also unable to calculate the cost-effectiveness of this program, lacking the medical cost data needed. A meta-analysis from 2010 [46], however, found modest but significant short-term savings for both medical costs (reductions of $3.27 for every dollar spent on wellness programs) and absentee day costs (reduced $2.73 for every dollar spent). A large study at Johnson and Johnson [47] found reduced medical costs for obese employees who reduced their weight as well as for nonobese employees who maintained their weight. This difference was particularly striking when contrasted with those employees who gained weight during the study time period, highlighting the importance of preventing weight gain. Our findings show that even employees who did not lose weight did show improved health behaviors such as increasing physical activity and fruit and vegetable consumption that could potentially prevent weight gain.

Despite these limitations, the present research contributes to the literature on the effectiveness and patterns of use of weight loss programs as delivered in the “real world.” Results presented here are indicative of the patterns of utilization of weight loss programs. Many people drop out of weight loss programs or fail to adhere to program requirements or to participate in program offerings. Despite this, a significant number of people may realize clinically significant weight loss and an even higher number may improve health behaviors, such as eating more fruits and vegetables and increasing physical activity, that can improve health generally even in the absence of clinically significant weight loss.

Further research is needed on the effectiveness of workplace applications of efficacious weight loss programs and related factors. For example, the literature could benefit from an examination of various methods of encouraging engagement and retention in workplace weight loss programs given the high numbers of individuals motivated to join a program but who drop out early on. Americans spend much of their time at work and workplaces have become increasingly sedentary over the past fifty years [48]. It is in the best interest of employers and society in general to promote methods of increasing the health of the workforce through intentional interventions such as the one described here.

## Figures and Tables

**Table 1:** Summary of website use.

	Mean	SD	Min	Max	Interquartile range		
					25%	50%	75%
Web logins	17.0	38.5	1	608	3	6	16
Activity tracker	13.9	55.4	0	1091	0	0	5
Step tracker	12.7	49.0	0	546	0	0	2
Weight tracker	5.1	12.5	0	312	1	2	6

**Table 2:** Logistic regression of ≥5% weight loss at 6 months by program use.

	OR	95% CI	*P* value
Number of coaching calls	1.16	[1.03, 1.30]	0.017
Number of website logins	1.01	[1.00, 1.01]	<0.001

Note: adjusting for age, gender, baseline BMI, baseline stress, and chronic disease history.

**Table 3:** Logistic regression predicting ≥5% weight loss at 6 months by web tools.

	OR	95% CI	*P* value
Total weight tracker uses	1.04	[1.02, 1.07]	0.002
Total activity tracker uses	1.00	[1.00, 1.01]	0.428
Total step tracker uses	1.00	[1.00, 1.00]	0.460

Note: controlling for age, gender, baseline BMI, baseline stress, and number of coaching calls.

**Table 4:** Logistic regression predicting ≥5% weight loss by health behavior changes.

	OR	95% CI	*P* value
Fruit/vegetable increase	1.45	[1.08, 1.94]	0.013
Breakfast increase	1.35	[1.00, 1.81]	0.047
Physical activity increase	1.38	[1.05, 1.83]	0.022
Stress decrease	1.53	[1.07, 2.19]	0.021

Note: controlling for age, gender, baseline BMI, and baseline stress.
